# Comparing the Neuropsychiatric Profile of Patients with Alzheimer Disease Who Present Spared versus Impaired Executive Functioning

**DOI:** 10.1155/2011/514059

**Published:** 2011-10-26

**Authors:** Ezequiel Gleichgerrcht, Anabel Chade, Teresa Torralva, María Roca, Facundo Manes

**Affiliations:** ^1^Cognitive Neuroscience Laboratory, Institute of Cognitive Neurology (INECO), Pacheco de Melo 1860, 1126 Buenos Aires, Argentina; ^2^Laboratory of Cognitive Neuroscience, Universirty Diego Portales, 8370179 Santiago, Chile; ^3^Institute of Neurosciences, Favaloro University, 1078 Buenos Aires, Argentina

## Abstract

*Background*. A “dysexecutive” group of patients with Alzheimer disease (AD) has been previously identified, and these patients have been found to present higher frequency of psychiatric symptoms and more pronounced functional impact. This study aimed at evaluating the frequency of neuropsychiatric symptoms in patients with early AD who present with impaired executive functioning. *Methods*. Thirty patients with early AD diagnosis were divided into a spared (SEF) and an impaired (IEF) executive functioning group according to their performance scores on neuropsychological tests. Their closest relatives or caregivers completed the Cambridge behavioral inventory (CBI), which assesses behavioral symptoms grouped into 13 categories. *Results*. A significant difference was exclusively found between SEF and IEF in terms of the frequency of stereotypies and repetitive motor behavior (*U* = 60.5, *P* = .024). *Conclusions*. The presence of stereotypies could be associated with a dysexecutive profile in AD patients. These results shed light on the role of frontal circuitry in the expression of motor symptoms in AD and prompt for further research that will contribute to the differential diagnosis both of different subtypes of AD and other types of dementia.

## 1. Introduction

The identification of a subgroup of Alzheimer disease (AD) patients who present with prominent frontal lobe dysfunction has gained increasing evidence in the last decade. While it is recognized that the involvement of the frontal lobes in AD patients is more subtle than in other degenerative diseases, such as frontotemporal dementia (FTD), several studies have shown that executive dysfunction occurs even in the early stages of the disease [1–4]. This “frontal” group of AD patients appears to have distinct neuropathological [5] and biochemical [6] changes within the frontal cortex, as well as characteristic neuropsychological profiles [7, 8] relative to patients with “typical” AD who develop the characteristic features of the disease as expected throughout time, including early prominent memory impairment. In fact, it has been recently suggested that disease progression in this subgroup may be more rapid than in AD patients of the amnesic (i.e., “typical”) type [7]. 

From a clinical and functional standpoint, AD patients with marked dysexecutive functioning tend to present higher frequency and severity of neuropsychiatric symptoms [2, 9–11] and decreased performance on activities of daily living [2]. Most frequently, studies that have screened for neuropsychiatric disturbances in AD have employed the neuropsychiatric inventory (NPI) [12] or its brief counterpart NPI questionnaire (NPI-Q) [13], both of which are carer-based interviews useful in everyday clinical practice because of its succinct nature. Another useful tool in the assessment of neuropsychiatric and behavioral impairment is the Cambridge behavioral inventory (CBI) [14], an informant-based 81-item questionnaire grouped into thirteen functional/behavioral domains, including memory, orientation and attention, everyday skills, self-care, mood, beliefs, challenging behavior, disinhibition, eating habits, sleep, stereotypic and motor behavior, motivation, and insight/awareness. Each item is rated on a scale from 0 (never) to 4 (constant occurrence) depending on the frequency with which the patient exhibits each particular behavior. 

While the NPI was developed in order to capture the neuropsychiatric symptoms of patients with dementia of the Alzheimer type [12], the CBI was originally designed to detect the behavioral disturbances that occur in patients with frontal variant FTD [14]. In this sense, it can be both useful and informative to assess “frontal” AD patients with a comprehensive questionnaire that taps on multiple behavioral domains typically affected in patients characterized by involvement of the frontal lobes (i.e., FTD patients). 

In the present study, the CBI was used to compare the behavioral profile of AD patients who presented spared executive functions relative to those patients with AD who exhibited marked dysexecution. We hypothesized that “frontal” AD patients would display higher frequency of neuropsychiatric symptoms than “typical” AD patients, in particular, for behavioral domains associated with frontal lobe functioning (e.g., disinhibition).

## 2. Methods

### 2.1. Participants

Patients with diagnosis of AD were recruited for this study according to the following inclusion criteria: (a) they fulfilled diagnostic criteria according to NINCDS-ADRDA consensus [15], (b) were in the mild stages of the disease, as determined by a clinical dementia rating scale (CDR) score of 1 [16], (c) presented medial temporal atrophy on MRI, and (d) signed an informed consent together with their caregiver prior to inclusion in the study. All patients completed the Beck depression inventory II (BDI-II) [17] in order to determine the severity of mood symptoms. A total of 30 AD patients matched these criteria and were included in the present study together with their closest caregiver. Caregivers were sons/daughters (73.3% of the cases), nieces (6.66%), or professional caregivers (20%). It was determined that the CBI domains had a high interrater reliability coefficient (mean Cohen's kappa =  .90; range:  .87 to  .95), as calculated from the cases (30%) for which more than two independent caregiver reports were available (e.g., that of the patient's son and professional caregiver).

### 2.2. Procedure

The study was initially approved by the ethics committee at the Institute of Cognitive Neurology (INECO, Buenos Aires, Argentina) in accordance with the guidelines established under the Declaration of Helsinki for research with human subjects. Following initial neurological interview, during which the aforementioned inclusion criteria were confirmed by a specialist cognitive neurologist (FM) for all participants, patients were assessed by a neuropsychologist with the Addenbrooke's cognitive examination—Revised (ACE-R) [18], a screening tool to assess general cognitive status and four classical tests of executive functioning, including working memory with the backward digit span (BckDS) test [19], phonological fluency (PhFlu) with the number of items produced with letter “P” in one minute [20], set-shifting with the trail making test part B (TMT-B) [21], and flexibility and abstraction capacity with the modified version of the Wisconsin card sorting test (WCST) [22]. Patients' caregivers were asked to complete the CBI, which was not used for diagnostic purposes so as to avoid circularity of our findings. Patients who were receiving antipsychotic medication were excluded from this study.

### 2.3. Statistical analysis

Individual raw scores on the four classical executive tasks (BckDS, PhFlu, TMT-B, and WCST) were initially transformed into *z*-scores according to available normative data for age- and gender-matched samples. AD patients who showed *z* scores ≤−1.5 on none or one of the four executive tasks were classified into the spared executive functioning AD group (SEF). On the contrary, AD patients showing *z* scores ≤−1.5 on two or more executive tasks were grouped into the impaired executive functioning AD group (IEF). CBI domain subscores were determined for each of the thirteen groups of symptoms as the sum of the individual frequency scores for each item included in each particular domain. 

Demographic variables, neuropsychological performance, and behavioral scores were compared between the groups using Student's *t*-test, or Mann Whitney *U* test when equal variances could not be assumed. For categorical variables (e.g., gender), a *χ* ^2^ test was applied. Correlations between variables were analyzed through Spearman's coefficients.

## 3. Results

Following the patient grouping criteria detailed in the Methods section, 19 AD patients were classified into the SEF group and 11 AD patients into the IEF group. As shown in Table 1, no significant differences were found between the groups on age (*t*_28_ = −2.06, *P* = .47), gender (*χ*_1_^2^ = .003, *P* = .96), dementia severity as measured by the CDR transformed score (CDR-TS, *t*_28_ = −.81, *P* = .76) and the CDR sum of boxes (CDR-SOB, *t*_28_ = −.73, *P* = .81), or the severity of mood symptoms (*t*_28_ = −.51, *P* = .82). A significant difference was found between the groups on the total score of the ACE-R (*t*_28_ = 4.55, *P* < .001), most likely associated with the poorer executive performance observed in the neuropsychological tests.

**Figure 1:**
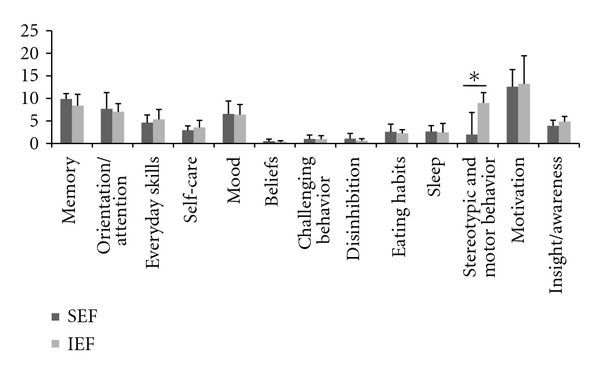
Mean (SD) frequency subtotal for the thirteen domains assessed by the Cambridge Behavioral Inventory. A significant difference was observed only for stereotypic and motor behavior (*P < .05).

As shown in Figure 1, a comparison of domain subtotal scores between SEF and IEF revealed no significant differences on the frequency of memory (*U* = 73.0, *P* = .19), orientation and attention (*U* = 89.0, *P* = .53), everyday skills (*U* = 93.5, *P* = .64), self-care (*U* = 88.0, *P* = .50), mood (*U* = 75.0, *P* = .22), beliefs (*U* = 87.0, *P* = .47), challenging behavior (*U* = 100.0, *P* = .87), disinhibition (*U* = 78.0, *P* = .27), eating habits (*U* = 88.5, *P* = .50), sleep (*U* = 80.0, *P* = .31), motivation (*U* = 140.0, *P* = .98), and insight/awareness (*U* = 72.5, *P* = .17). However, a significant difference was found between the groups on the stereotypic and motor behavior frequency of symptoms (*U* = 60.5, *P* = .024). 

When patients were considered altogether, no significant correlations were found between scores on the CBI subdomains and neuropsychological performance on either the ACE-R or any of the four executive tasks, except for a significant positive association between the CBI's motivation domain and performance on the WCST (*r* = .42, *P* = .02). Within the SEF group alone, the total ACE-R score was significantly and negatively correlated with the Memory (*r* = −.42, *P* = .05) and the Orientation/Attention domains of the CBI (*r* = −.46, *P* = .04). Within IEF patients, performance on the WCST (*r* = −.56, *P* < .01) and the TMT-B (*r* = −.43, *P* = .03) correlated significantly with the Stereotypies domain of CBI. No other significant correlations were found.

## 4. Discussion 

Our results revealed that AD patients who presented impaired executive functioning exhibit significantly more stereotypies and repetitive motor behaviors than patients with AD who show spared performance on this domain. No other significant differences were found on any of the other neuropsychiatric/behavioral domains assessed by the CBI. 

Bozeat et al. [14] had demonstrated that of all domains in the CBI, stereotypic behavior was one of the few that reliably differentiated patients with AD from behavioral variant frontotemporal dementia. Taking into account the selective involvement of the ventromedial prefrontal cortex in the latter condition during the earlier stages of the disease and considering the marked neuropathological changes that have been described within the frontal cortex of “frontal” AD patients [5], it comes as no surprise that stereotypic and aberrant motor behavior—which strongly depend on frontal cortex activity—were more prominent in dysexecutive AD patients than in those who show spared executive functions. In this sense, the results of this preliminary study support the idea of a frontal subgroup of AD patients who may have more involvement of the frontal lobes [5, 6] and hence exhibit more marked neuropsychiatric and behavioral disturbances [2, 9, 11, 23] associated with the circuitry within the anterior regions of the brain. 

Other behavioral domains assessed by the CBI, however, have also been strongly associated with the frontal cortex. For example, studies employing voxel-based morphometry have related apathy, abnormal eating, and disinhibition with ventromedial frontal cortices [24, 25]. The question then remains: why was a significant difference found particularly for the domain of stereotypic behaviors? Although not without controversy, one could argue that—even if behavior obviously results from integrated networks—stereotypic behavior depends more strongly on frontal striatal components [26] while apathy and disinhibition rely more strongly on frontal cortical regions [27]. One possibility that must be studied in similar studies using larger samples is that the underlying pathological changes within “frontal” AD patients could affect striatal—rather than cortical—aspects of the frontal circuitry more prominently, or else, earlier in time. The description of frontal neuropathological patterns within this subgroup of AD patients is relatively new and has been done at the level of big brain regions (e.g., “frontal cortex”) [5, 6], but studies looking at structural and biochemical changes of different subnetworks within a brain area may shed light on the heterogeneous nature of Alzheimer disease. As other authors have recently pointed out, the differentiation of a frontal variant of AD is clinically possible but it still requires pathological verification [28]. Ideally, thus, future studies should document neuropathological changes separately for different components of the frontal corticosubcortical loops (e.g., cortical versus striatal changes). Specifically, if cortical (versus subcortical) regions of the frontal lobes are spared until later in the progression of “frontal” AD patients, stereotypic behavior may be the result of early frontal striatal involvement. It must be noted that all AD patients in the present study were in the mild stages of the disease. A crucial issue for future studies will be also to compare “frontal” AD patients in the mild, moderate, and advanced stages of the disease to determine whether different neuropsychiatric profiles emerge at different stages of the disease. Remarkably, general cognitive status, as measured by the ACE-R, was associated with memory and attention/orientation scores on the CBI within the group of AD patients with spared executive functioning. Among those who had impairments in this domain, however, a negative association was found between executive functioning (TMT-B and WCST) and the frequency and severity of stereotypies. This could also suggest that, at least within “frontal” AD patients, executive functioning—which depends strongly on the prefrontal cortex and its striatal connections—may be related with behavioral disturbances associated with this brain region. If this is indeed the case, rehabilitation programs focused on executive functioning could also result in enhanced behavior. 

By incorporating a stereotypies-specific addendum to the NPI, Nyatsanza et al. [29] had already shown that, while complex ritualized behaviors were more frequent in patients with frontal variant frontotemporal dementia and semantic dementia, simpler stereotypies were equally common in the AD population. For this reason, future studies should also try to determine whether the nature of increased stereotypies in “frontal” AD patients features more complex behaviors than those exhibited by the “typical” AD group, as the former would then mimic more closely those behaviors found in “frontal” conditions. Evidence derived from studies of this nature will contribute to the development of more efficient differential diagnostic strategies both for AD and for FTD. Patients with AD who exhibit atypical frontal involvement could be potentially misdiagnosed as FTD, thus delaying early pharmacological and nonpharmacological interventions that may be useful for AD but not for FTD. By the same token, FTD patients presenting with severe amnesia could be as well misdiagnosed with AD [30]. In this sense, while the NPI has been used to assess patients with FTD [31, 32] and the CBI to assess patients with AD [33], combining the two tools may be helpful for cases of patients whose behavioral and cognitive disturbances make differential diagnosis especially challenging. With recent studies supporting the idea that the frontal subgroup of AD patients may be indeed clinically distinguished from other AD patients [28], future studies on this issue must also compare stereotypies and motor symptoms with those exhibited by FTD patients to help develop strategies to distinguish frontal AD patients from FTD, and amnesic FTD from AD. 

The present study has two obvious limitations. On the one hand, the relatively small sample size of this study demands that the findings hereby presented be replicated in larger populations, and as stated above, in different stages of the disease. By doing so and by correcting for multiple comparisons, we will be able to draw stronger inferences about the relatively selective deficit in stereotypic behaviors among “frontal” AD patients. On the other hand, no pathological confirmation of AD diagnosis is available for patients included in this study. In this respect, it must be noted that patients were longitudinally followed (between 3 and 6 years), and they all went on to exhibit typical symptoms and progression of AD. As well, to date, there are unfortunately no universally accepted criteria to determine what constitutes “executive dysfunction”. Our cutoff of more than one executive task with performance *z* score <−1.5 SD was based on previously published procedures [8]. 

Besides these caveats, the present results have important clinical implications that derive from the issues mentioned above. First, they reveal the need to assess neuropsychiatric and behavioral changes in patients with dementia, as they have the potential to provide important information about the challenges faced by patients and their relatives on an everyday basis. Second, it prompts for the development of histopathological techniques that will allow for the characterization of structural and biochemical changes within subcircuits of different brain areas, therefore providing more detailed accounts of pathological changes in different components of complex brain circuits. Third, our findings highlight the importance of assessing domains known to rely on frontal circuitry, such as stereotypic behaviors for various reasons. On the one hand, because marked behavioral disturbances that are perceived by others as odd can be extremely burdensome for relatives of patients with dementia, understanding the intricate details of abnormal behaviors can also help in educating relatives and caregivers about how to cope with such symptoms. On the other, it can contribute to the differential diagnosis of potential AD subtypes and, furthermore, between different types of dementia, such as FTD. In doing so, the field will move towards more personalized treatment plans by employing relevant and *ad hoc* pharmacological and nonpharmacological strategies to each subpopulation within heterogeneous patient groups.

## Figures and Tables

**Table 1:** Comparison of demographic variables and neuropsychological performance for SEF and IEF patients.

	SEF (*n* = 19)	IEF (*n* = 11)
Age	76.4 (6.4)	78.2 (4.3)
Gender (M : F)	9 : 10	5 : 6
Age at onset	74.1 (8.4)	76.1 (2.6)
CDR-TS	.66 (.29)	.86 (.43)
CDR-SOB	6.2 (1.3)	7.8 (1.5)
BDI-II	5.9 (2.3)	6.4 (2.4)

ACE-R total score	74.8 (9.0)	58.0 (10.3)

Phonological fluency *(no. of words/min) *	11.8 (3.2)	8.82 (3.3)
Backward digit span * (no. of digits repeated) *	3.59 (1.2)	2.5 (1.0)
Trail Making Test Part B * (seconds to complete) *	182 (97)	343 (55)
Wisconsin Card Sorting Test * (no. of correct categories) *	3.00 (1.8)	2.33 (2.0)

CDR: clinical dementia rating scale, CDR-TS: CDR transformed score, CDR-SOB: CDR sum of boxes, BDI-II: Beck depression inventory II, ACE-R: Addenbrooke's cognitive examination—revised.
